# Back to beaked: *Zea mays* subsp. *mays* Rostrata Group in northern Italy, refugia and revival of open-pollinated maize landraces in an intensive cropping system

**DOI:** 10.7717/peerj.5123

**Published:** 2018-07-04

**Authors:** Nicola Maria Giuseppe Ardenghi, Graziano Rossi, Filippo Guzzon

**Affiliations:** Department of Earth and Environmental Sciences, University of Pavia, Pavia, Lombardia, Italy

**Keywords:** Ethnobotany, Agrobiodiversity, Plant genetic resources, Custodian farmers, Open pollinated varieties

## Abstract

Crop landraces are fundamental resources to increase the eroded genepool of modern crops in order to adapt agriculture to future challenges; plus, they are of immeasurable heritage and cultural value. Between the 1940s and the 1960s open-pollinated varieties (OPVs) of flint and semi-flint maize in Europe were almost completely replaced by high-yielding hybrid dent cultivars selected in North America. No comprehensive assessment was performed after the 1950s to understand which maize genetic resources survived genetic erosion in northern Italy, an area characterized by a high degree of landraces extinction and introgression, intensive hybrid dent monocultures, as well as being one of the hotspots of maize cultivation at a continental level. Among these landraces, beaked maize represents a peculiar case study for assessing the survival of OPVs in intensive cropping systems. By means of ethnobotanical and literature surveys, the history of *Zea mays* subsp. *mays* Rostrata Group and its current distribution were reconstructed. It emerged that beaked maize originated in the study area and it is one of the oldest genepools available not subjected to formal crop improvement. We identified 28 landraces of beaked maize currently cultivated, 18 here recorded for the first time. The cultivation of more than half of the 28 landraces has continued throughout the last 80 years in a few fragmented localities that can be regarded as “refugia”. The survival of these landraces from substitution with high-yielding cultivars and unidirectional introgression has been mainly due to active on-farm conservation performed by custodian farmers and secondarily to cultivation in isolated areas (e.g., mountain valleys). After decades of genetic erosion, beaked maize has since the late 1990s experienced a revival, in terms of an increasing number of cultivation localities and the level of product commercialization. This process is mostly spontaneous and only occasionally mediated by governmental institutions; it is linked to the rediscovery of local food products, in this case mainly polenta, a dish made of corn flour, which used to be the staple food across northern Italy. The *ex situ* conservation of beaked maize and on-farm measures put in place by the farmers to prevent introgression are also assessed. Further research and collecting missions are needed to provide an inventory of open-pollinated landraces of other landrace groups that have survived genetic erosion in Europe. To meet this aim, extensive ethnobotanical surveys, such as the one performed here, are very powerful tools in detecting these genetic resources.

## Introduction

Crop genetic diversity is an indispensable resource for farmers and breeders to select new crop cultivars (McCouch et al., 2013; Dyer et al., 2014). The bulk of genetic diversity in crops is found in landraces (Camacho Villa et al., 2006; Zhou et al., 2015), defined as: “plant materials consisting of cultivated varieties that have evolved and may continue evolving, using conventional or modern breeding techniques, in traditional or new agricultural environments within a defined ecogeographical area and under the influence of local human culture” (Casañas et al., 2017). Additionally, crop landraces often show interesting adaptations towards marginal environments and pest resistance (Camacho Villa et al., 2006; Mikami, Carpentieri-Pípolo & Ventura, 2012; Mabhaudhi et al., 2016; Guzzon et al., 2017). For these reasons, landraces are of key importance in increasing the eroded genepool of modern cultivars with the aim of adapting agriculture to climatic changes and achieving a more resilient and sustainable agricultural system (Veteläinen, Negri & Maxted, 2009; Warschefsky et al., 2014). Not only do landraces possess useful traits for crop breeding, but they are of immense heritage value, linked to local and traditional products, and should be preserved for their importance in enhancing food sovereignty and safeguarding cultural diversity (Pieroni, Pawera & Shah, 2016; Casañas et al., 2017; De Luca et al., 2018).

The genetic and cultural wealth of landraces is threatened by their replacement, but also by introgression with modern cultivars (Bitocchi et al., 2009; Scholten et al., 2012). Over the last few decades, in several areas of the world severe genetic erosion of crops has taken place leading to a loss of more than 70%, in terms of crop genotypes and accessions (Hammer et al., 1996; Veteläinen, Negri & Maxted, 2009; Van de Wouw et al., 2010). In order to avoid landrace extinction, conservation strategies, including complementary *in situ* and *ex situ* conservation measures, must be put in place (Altieri & Merrick, 1987; Negri & Tiranti, 2010). The prerequisite of on-farm conservation programmes is on the availability of inventories of landraces. These inventories are fundamental to designing national and regional conservation strategies and for monitoring the efficiency of conservation actions (Negri, Maxted & Veteläinen, 2009; Casañas et al., 2017). Moreover, landrace inventories are needed to understand the geographical pattern of landrace distribution which helps to guide the collecting missions of plant genetic resources (Perales & Golicher, 2014; Orozco-Ramírez, Perales & Hijmans, 2017). According to some sources, over the last few years, in several European countries a process of reviving landraces has been instigated by farmers and consumers, associated with traditional food production and the cultivation of marginal areas (Koutsika-Sotiriou et al., 2010; Barthel, Crumley & Svedin, 2013; Casañas et al., 2017). Nonetheless, this process is not homogenous and it is likely to occurr only for some crops and/or in some areas, while in other situations abandonment of traditional genetic resources and the substitution of landraces with high-yielding modern cultivars are still major threats for agrobiodiversity conservation (Teklu & Hammer, 2006; Dyer et al., 2014; Quave & Saitta, 2016; Ardenghi et al., 2017).

Maize (*Zea mays* L. subsp. *mays*) cultivation in Europe appears to be as a very suitable model for the study of all the aforementioned phenomena of agrobiodiversity loss, conservation and revival. Europe is the third largest maize producer and consumer worldwide (Bitocchi et al., 2009). Maize firstly arrived in Europe from the Americas with the voyages of Christopher Columbus. After the first introduction of Caribbean samples, subsequent introductions of maize germplasm from higher altitudes and latitudes in the Americas better adapted to European conditions, especially to longer photoperiods, boosted maize cultivation in Europe. Since then, a multitude of landraces linked to local food production and traditional farming systems have been developed, through crosses of different ecotypes, by human selection and through adaptations to different environmental conditions (Brandolini & Brandolini, 2009). Northern Italy is one of the cores of European maize cultivation and production, with a harvested production of grain maize and corn-cob mix of more than 6 million tons per year. Two administrative regions, Lombardia and Veneto, exceeded 1.7 million tons per year (Eurostat, 2018). In most of northern Italy, until the 20th century, flint corn was the staple crop, connected with the cooking of polenta, a porridge-like traditional dish made using boiled maize flour. Similar to other European areas, after the Second World War, open-pollinated varieties (OPVs) of flint and semi-flint corn were almost completely replaced, by the end of the 1960s, by high-yielding hybrid dent cultivars selected in North America (Brandolini & Brandolini, 2006; Brandolini & Brandolini, 2009; Bitocchi et al., 2009). Genetic erosion in northern Italy is reported by Hammer et al. (1996) to have reached more than 90% in the last few decades. Researchers recognized the genetic erosion that was occurring in the maize genepool and organized collecting missions in 1949 and 1954, followed by classification studies of the germplasm collected. The accessions collected from those missions, which are still conserved by the Unità di Ricerca per la Maiscoltura (CREA-MAC) of Bergamo (Lombardia, Italy), give a snapshot of the maize landrace occurrence in Italy just before the complete switch of maize cultivation from mixed subsistence farming units to intensive monocultures of hybrid dent cultivars.

Among the most characteristic accessions collected was the beaked maize, vernacularly known as “*Rostrato*” (i.e., “beaked”). This assemblage of landraces, whose history has never been unambiguously clarified, was thought to have originated in northern Italy and for a period was preferred by farmers to other flint landraces for the production of polenta. It belongs to an early generation of local open-pollinated landraces that preceded the introduction of the improved Italian cultivars in the 1920s–1930s and the United States’ dent hybrids in the late 1940s (Lanza, 1961; Brandolini & Brandolini, 2001; Brandolini & Brandolini, 2006; Brandolini & Brandolini, 2009).

Even though no comprehensive surveys were performed in northern Italy to assess maize landraces that might have survived genetic erosion after the 1950s, some authors suggest that a few flint maize landraces still survive in remote valleys in the Alps and Apennines, cultivated by amateur farmers (Bitocchi et al., 2009; Brandolini & Brandolini, 2009). The conservation of flint maize landraces in Europe is considered of pivotal importance since OPVs may possess allelic variations not yet used in the selection of elite corn cultivars (Reif et al., 2005). The on-farm conservation of OPVs, in areas characterized by monocultures of hybrid dent corn, is particularly threatened by unidirectional gene flow from modern cultivars to landraces which could result in the genetic extinction of the latter (Bitocchi et al., 2009; Brandolini, 1970). On the other hand, it is interesting to note that some OPVs in southern Europe are going through a process of rediscovery by farmers and consumers thanks to their peculiar organoleptic characteristics and richness in anthocyanins, such as ‘Millo Corvo’ in Spain and ‘Nero Spinoso’ and ‘Scagliolo di Carenno’ in northern Italy (Lago et al., 2014; Lago et al., 2015; Cassani et al., 2017).

In this paper, through ethnobotanical surveys across all northern Italian provinces and an extensive literature review, besides an in-depth historical account and a clear-cut description of *Zea mays* subsp. *mays* Rostrata Group and allied crosses, we provide the first inventory of all beaked maize landraces that can currently be found in the study area, along with the assessment of their *ex situ* conservation status and the measures put in place on-farm by the farmers to prevent or limit introgression events with hybrid dent corn. Moreover, for each landrace we take into consideration elements associated with its history, cultivation practices and geographical location, with the aim of understanding if its current localities of cultivation can be recognized as “refugia”, i.e., restricted areas provided with a combination of environmental and human characteristics that allowed the survival of these landraces in the course of the last century. Furthermore, we have evaluated whether a “revival” process, in terms of increasing agronomic and economic interests, is occurring for the Rostrata Group consistent with what has been found for other OPVs in Europe (see e.g., Lucchin, Barcaccia & Parrini, 2003; Lago et al., 2014; Cassani et al., 2017).

## Survey Methodology

### The study area

**Figure 1:**
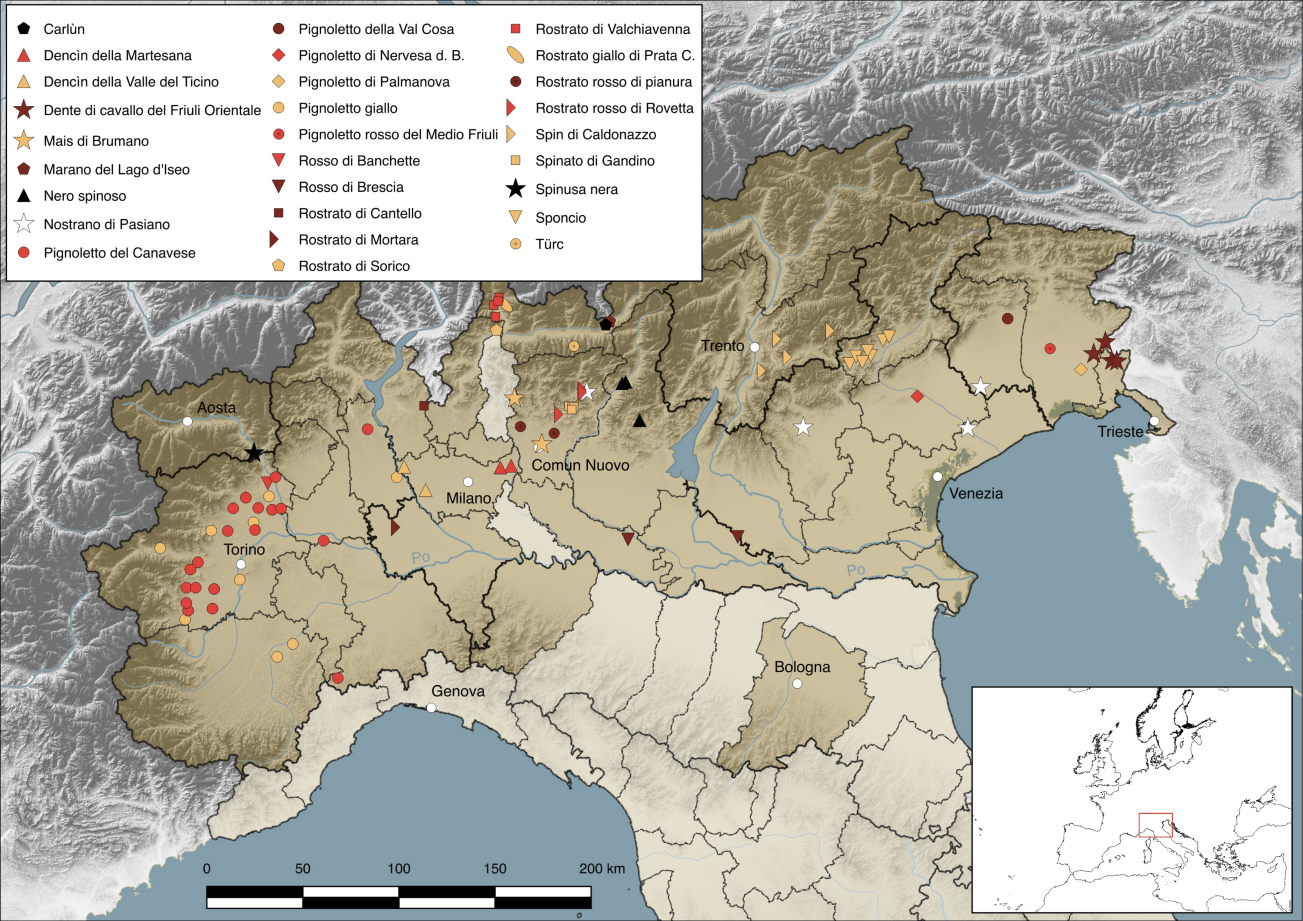
Study area. The location of each present beaked maize landrace and allied cross is marked by means of a different symbol (see also Table S1). Boundaries of regions are thicker and continue, those of provinces are thinner and dashed; regional administrative centres has been highlighted as well the municipality of Comun Nuovo. The administrative provinces where the presence of beaked maize landraces and their crosses has been recorded either in historical (pre-1990s) or contemporary (post-1990s) times are brightly colored. Map credits: Nicola M.G. Ardenghi.

The study area comprises all the administrative provinces of northern Italy where the cultivation of beaked maize has been reported in the past (pre-1990s) and present (post-1990s) times. These, together with the municipalities where the cultivation of beaked maize persists today, are highlighted in Fig. 1 and listed in Table S1. For each municipality, we specified in Table 1 the associated altitudinal range (“plain”: <300 m a.s.l.; “hill”: 300–600 m a.s.l.; “mountain”: >600 m a.s.l.), based on Istat (2018).

**Table 1:** Investigated landraces and associated names, geographical and altitudinal distribution, and historical information. Landrace names partly or completely inserted between square brackets were coined by the authors. The complete list of the municipalities where each landrace is cultivated is reported in Table S1. Each altitudinal range is indicated by means of the following letters: “P”, plain; “H”, hill; “M”, mountain. Sources investigated for each landrace are reported in Table S1.

**Code**	**Landrace name**	**Synonym(s)**	**Distribution (region: *province*)**	**Altitudinal range %**			**Historical notes**
				**P**	**H**	**M**	
R1	Dencìn [della Martesana]	–	Lombardia: *Milano*	100	0	0	Cultivated by the Rolla family in Cernusco sul Naviglio and in the Martesana (Milano) and Bassa Brianza (Monza e Brianza) areas until 1960–1965. Its cultivation was recovered in 2005 from two ears discovered by A. Rolla in a barbershop in Bellinzago Lombardo (Milano).
R2	Dencìn or Scagliolo [della Valle del Ticino]	–	Lombardia: *Milano*	100	0	0	Obtained by the Passerini brothers (Cascina Cirenaica, Robecchetto con Induno) in 2001 from an old farmer at a local fair; it was subsequently donated to another farmer (Azienda Aia, Cassinetta di Lugagnano) in 2013. Their father cultivated another beaked maize in the Nerviano-Rho (Milano) area.
R3	Dente di cavallo [del Friuli Orientale]	–	Friuli-Venezia Giulia: *Gorizia*, *Udine*	100	0	0	A landrace belonging to the Rostrato × Dentato Group, it is traditionally cultivated in the Italian portion of the Friuli Orientale area and, at least in the past, also beyond the present border with Slovenia. Both white and red kernels are sown, yet only white are used to produce the maize flour (which is white).
R4	[Mais di Brumano]	–	Lombardia: *Bergamo*	50	0	50	Cultivated since the 1980s in Brumano by G. Pirola (who later donated the seeds to a farmer in Zanica); it originated in Pirola’s fields from the spontaneous crossing of a yellow flint landrace from Trentino and a red-beaked semi-flint landrace from Valtellina.
R5	Nero spinoso	Mèlga negra spinúsa, Rostrato di Esine, Spinato di Esine, Spinùs	Lombardia: *Brescia*	0	0	100	Cultivated by the Saloni family in Annunciata (Piancogno) since the early 20th century; its cultivation was locally revived in 2015. Introduced in Pertica Alta around 2010, where a line (named “Spinùs”) provided with red kernles is being selected.
R6	Nostrano di Pasiano	Blave dente di cavallo	Friuli-Venezia Giulia: *Pordenone*; Lombardia: *Bergamo*; Veneto: *Venezia*, *Vicenza*	75	0	25	A member of the Rostrato × Dentato Group, it has been cultivated since 2017 by D. Pizzolato in Marano Vicentino; seeds were acquired in 2016 from Luigi Piccinin of Pasiano di Pordenone, who sows both red and white kernels. It is still cultivated in other parts of Friuli-Venezia Giulia and by a miller in Noventa di Piave. Its cultivation was also started by a farmer in Songavazzo (thanks to seeds distributed by D. Pizzolato), to provide restaurants in the Lake Iseo area with white maize flour.
R7	Pignoletto del Canavese	Pignòlet, Pignoletto rosso, Rostrato dente di cane	Piemonte: *Alessandria*, *Novara*, *Torino*, *Vercelli*	47.4	52.6	0	Cultivated mainly in the subalpine Canavese area (Torino) from circa 1920s–1930s until the mid 1960s, then largely abandoned. Its cultivation was revived in 2002 by the Province of Torino’s Centro di Riferimento per l’Agricoltura Biologica (CRAB) with germplasm acquired from Valperga (Torino).
R8	Pignoletto della Val Cosa	–	Friuli-Venezia Giulia: *Pordenone*	0	100	0	Cultivated by G. Lenarduzzi in Sequals since circa 2007; seeds were acquired from a local farmer.
R9	Pignoletto [di Nervesa della Battaglia]	–	Veneto: *Treviso*	100	0	0	Cultivated since 2015 by M. Celotto in Nervesa della Battaglia; seeds were acquired from a farmer in the area of the River Cellina (province of Pordenone, Friuli-Venezia Giulia).
R10	Pignoletto [di Palmanova]	Pignoletto giallo	Friuli-Venezia Giulia: *Udine*	100	0	0	Seeds handed down from generation to generation.
R11	Pignoletto giallo	Pignòlet, Pignoletto del torinese	Piemonte: *Cuneo*, *Novara*, *Torino*	56.3	37.5	6.3	Cultivated mainly in the subalpine Canavese area (Torino) from circa 1920s–1930s until the mid 1960s, then largely abandoned. Its cultivation was revived in 2002 by the Province of Torino’s Centro di Riferimento per l’Agricoltura Biologica (CRAB) with germplasm acquired from Alpignano (Torino).
R12	Pignoletto rosso [del Medio Friuli]	–	Friuli-Venezia Giulia: *Udine*	100	0	0	Traditionally cultivated in the Medio Friuli area, its cultivation has been revived in recent times.
R13	Rosso di Banchette	Pignoletto rosso di Banchette	Piemonte: *Torino*	0	100	0	Its cultivation was revived in 2005 by a group of farmers from Banchette (association “Biocolture Banchette”).
R14	Rosso di Brescia	–	Lombardia: *Cremona*, *Mantova*	100	0	0	Cultivated across the provinces of Brescia (plain area), Cremona, and Mantova from the late 19th century until circa 1960s. Its cultivation started in 2010 by Emanuela Dilda in Pessina Cremonese, from seeds acquired around 2008 in Val Camonica and donated by E. Amadio, teacher at the Istituto Agrario Stanga of Cremona.
R15	Rostrato di Cantello	–	Lombardia: *Varese*	0	100	0	Originally collected from Cantello by the Stazione di Maiscoltura of Bergamo (now CREA-MAC) in 1963; seeds obtained from this institution were used to recover its cultivation in Cantello in 2015 by Federica Baj (Bio Baj).
R16	[Rostrato di Mortara]	–	Lombardia: *Pavia*	100	0	0	Recovered by G. Manzini from ears discovered in his family’s farm in Mortara; it is cultivated since 2010. A strain provided with black and beaked kernles is being selected.
R17	Rostrato di Valchiavenna	–	Lombardia: *Sondrio*	0	0	100	Cultivated in Valchiavenna area since the early 20th century, its cultivation is locally being revived by Comunità Montana della Valchiavenna in collaboration with the University of Pavia.
R18	Rostrato rosso di pianura	Rostrato rosso dell’Isola	Lombardia: *Bergamo*	0	100	0	Cultivated in Ambivere and Albano Sant’Alessandro since 2010 and 2015 respectively; seeds were acquired from the nearby municipalities of Ciserano and Arcene.
R19	Rostrato rosso di Rovetta	Melgù, Melgòtt, Rampì, Rostrato Marinoni	Lombardia: *Bergamo*	0	0	100	Cultivated in Rovetta by the family of Giovanni Marinoni since the early 20th century; its cultivation was revived when some ears were detected in 2004 by agronomist Aureliano Brandolini in an ornamental basket at the festival of the Rovetta potato.
R20	Spin di Caldonazzo	Florian Red Flint, Nostrano della Valsugana	Trentino-Alto Adige: *Trento*; United States of America	0	0	100	Probably introduced from Veneto or southern Trentino (Ala, Arco, Riva del Garda) around the 1920s–1930s, it was cultivated in eastern Trentino on a large scale until the 1960s. Almost disappeared in subsequent years, its cultivation was locally revived in 2002.
R21	Spinato di Gandino	Melgotto	Lombardia: *Bergamo*	0	0	100	Its cultivation was recovered in the early 2010s from two ears discovered in 2008 in the Savoldelli family’s farm at Ca’ Parecia (Gandino).
R22	Spinusa nera	–	Valle d’Aosta	0	0	100	Cultivated by the Chappoz family in Donnas, it is an informal cross between ‘Isola’ (probably ‘Nostrano dell’Isola’ or a local derivative), cultivated in the first half of the 20th century in Donnas, and beaked landraces from near Viverone (Biella) and Borgofranco d’Ivrea (Torino) (locally known as ‘Spinusa’ and ‘Pignoletto’), introduced in Donnas around 1988. Three strains are obtained, respectively characterized by: muticous black kernels (unnamed, preferred by the farmers); beaked black kernels (‘Spinusa nera’); beaked pale kernels (‘Spinusa chiara’).
R23	Sponcio	Pignol, Pignol fiorentin, Rostrato	Veneto: *Belluno*	0	0	100	Known in Val Belluna since the late 19th century, its cultivation was revived in the late 1990s.
R24	Türc	–	Lombardia: *Sondrio*	0	0	100	Cultivated in Piateda by Mr. Mascarini since 2015; seeds were acquired locally.
R25	Carlùn	–	Lombardia: *Sondrio*	0	0	100	Cultivated by P. Moltoni in Villa di Tirano since 2003. Seeds were obtained from Cermenate (Como). “Carlùn” is a variant of “Carlón”, the vernacular name applied to maize in general in the Prealpine area of Lombardia and in Canton Ticino (Switzerland), traditionally but erroneously associated with the figure of Carlo Borromeo, archibishop of Milano from 1564 to 1584 (see Mariani, 2012).
R26	Marano [del Lago d’Iseo]	–	Lombardia: *Sondrio*	0	0	100	Despite its name, this landrace is not related with ‘Marano’. Cultivated by P. Moltoni in Villa di Tirano since circa 1998, it was obtained from a farmer of Costa Volpino (Bergamo), whose seeds were handed down from generation to generation.
R27	[Rostrato di Sorico]	–	Lombardia: *Como*	0	0	100	Local landrace, still cultivated in the municipality of Sorico.
R28	[Rostrato giallo di Prata Camportaccio]	–	Lombardia: *Sondrio*	0	0	100	Cultivated by M. Bonassoli and his mother from at least 2008. It originated from the spontaneous crossing of two landraces from Sorico: an unnamed flint maize with muticous yellow kernels (locally cultivated at least since 1970) and ‘Rostrato di Sorico’ (locally introduced around 2008).

Northern Italy consists of eight administrative regions (Emilia-Romagna, Friuli-Venezia Giulia, Liguria, Lombardia, Piemonte, Valle d’Aosta, Veneto, Trentino-Alto Adige) and 46 provinces covering an area of more than 120,000 square kilometres. This geographic area is characterized by wide alluvial plains, the Po and the Venetian Plains, enclosed by two main mountain ranges, the European Alps in the north and in the west and the Apennines in the south. The plain areas, thanks to the abundance of water, are particularly suited to large, industrial monocultures of maize and rice. Northern Italy is indeed a leading producer of the latter two cereals at a European level (USDA, 2012; FAO, 2017), with the area under maize totalling 894,452 ha (I.Stat, 2018).

Despite well-known genetic erosion, introgression events and substitution of landraces with “improved cultivars” recorded in the study area in a multitude of crop genepools, such as apple (see Breviglieri, 1949), maize (see Brandolini & Brandolini, 2009) and rice (see Giacosa, Rondanelli & Tinarelli, 2006), some genetic resources, in terms of landraces and neglected crops, previously unknown to the scientific community, were recently identified (see Ardenghi et al., 2017; Buffoli, 2015).

### Ethnobotanical and literature surveys

The present study is based on ethnobotanical surveys conducted by the authors across northern Italy (Fig. 1, Table S1). We located all the farmers involved in the cultivation of beaked maize landraces across the study area, thanks to personal acquaintance, by directly asking maize farmers if they were acquainted with landrace growers, by contacting researchers who deal with landrace conservation and cultivation in the study area, or simply by consulting newspapers, websites and social media network platforms. From these sources, we obtained a list of 35 beaked maize growers that were subjected to ethnobotanical interviews (mentioned in ‘Acknowledgements’) conducted between 2016 and 2018 face-to-face, by telephone, e-mail or through social networks. In order to obtain a uniform and comparable dataset, a unique semi-structured questionnaire was used in each interview. Questions concerned: (1) germplasm origin; (2) landrace history, distributional range, number of current growers and vernacular name; (3) cultivation, issues associated with true-to-type maintenance of the OPVs and products derived from them (Data S1). Verbal informed consent was received from each interviewed subject. Ears or shelled kernels donated from interviewed farmers were stored at the University of Pavia Germplasm Bank.

Information from interviews was integrated with data acquired from the investigation of all the available historical and contemporary agronomic, botanical and culinary literature sources (mentioned, for each landrace, in Table S1).

The combination of interviews and literature survey allowed the authors to trace, based on the best available knowledge, all landraces of beaked maize from the study area and to obtain data regarding the whole cultivation range of each investigated landrace.

### Terminology and nomenclature

This research is focused on the assemblage of landraces defined as Rostrata Group; in order to sample all of the genepool of this Group, crosses with other landraces have also been taken into account as well as members of the Rostrato × Dentato Group (see the “Identity” subheading).

The names of taxa (from family to variety ranks) and the names of culta (landraces, cultivars and Groups) are treated according to the rules of the International Code of Nomenclature for algae, fungi, and plants (ICN, McNeill et al., 2012) and the International Code of Nomenclature for Cultivated Plants (ICNCP, Brickell et al., 2016), respectively. In line with Zeven (1998), we distinguished on terminological grounds “landraces” from “cultivars”: although the term “landrace” is not treated by the ICNCP, its conception (as introduced by Zeven, 1998, and then updated by Casañas et al., 2017; see also ‘Introduction’), referring to entities “that have evolved or may continue evolving […] in traditional or new agricultural environments” (Casañas et al., 2017), is antithetic to that of “cultivar” that applies only to “uniform and stable” first-generation products of modern breeding (Hetterscheid & Brandenburg, 1995; Zeven, 1998). Regarding the several accessions found as a result of the ethnobotanical survey, we considered belonging to the same landrace a set of accessions with common geographical and historical origin, and locally known with the same vernacular names (in the few cases when the vernacular name was lacking, it was coined by the authors by combining a name recalling morphological traits of the landrace and its locality of provenance, see Table 1). More specifically, two or more accessions were considered as sharing a common origin when: (1) they derive from a single germplasm source in the time-interval of two generations of farmers; and/or (2) there is evidence of a constant gene flow between them in terms of seed swap among farmers cultivating the different accessions. Throughout the text, each landrace and cross has been cited by means of the associated alphanumeric code (see Table 1).

Morphological characteristics were designated by means of botanical terminology in the description of the Rostrata Group, whereas the related agronomical terms (such as “ears”, see Iltis, 2003; Kellogg, 2015; Spjut, 2015), reported between brackets in the description, are employed in other parts of this work.

### Conservation and revival

Insights into on-farm conservation were obtained through ethnobotanical interviews and literature searches, by considering whether and which actions are put in place by farmers to limit genetic introgression with dent hybrids. *Ex situ* conservation was assessed by checking the occurrence of seed samples of each investigated landrace in the databases of the major Italian and international genebanks devoted to maize germplasm preservation, e.g., Unità di Ricerca per la Maiscoltura (CREA-MAC) of Bergamo, Istituto Strampelli of Lonigo germplasm bank, University of Pavia Germplasm Bank, Svalbard Global Seed Vault and Trentino Seed Bank of MUSE—Museo delle Scienze di Trento, Trento (NordGen, 2010; PlantA-Res, 2013; Museo Tridentino di Scienze Naturali, 2018).

Based on information collected through ethnological and literature surveys we assessed the “revival” of the landraces. A landrace was considered revived when at least one of the following criteria was met: (1) its cultivation was resumed or increased in terms of the number of localities of cultivation in the last 20 years; (2) products derived from the landrace that have started to be commercialized to the public in the last 20 years; (3) occurrence of the following protection and promoting measures applied to the landrace itself or its products (see also Porfiri, Costanza & Negri, 2009): (a) National Register of Conservation Varieties: inventory of the Italian conservation “varieties” (i.e., cultivars and landraces) established by the Italian Ministry of Agricultural, Food and Forestry Policies (Law 6 April 2007, n. 46) in order to adopt European Union Directives (see e.g., Commission Directive 2003/90/CE of 6 October 2003); (b) Slow Food Presidia: promotional brand assigned to traditional products, processing methods and landscapes at risk of extinction by Slow Food, international organization for the preservation of local food cultures and traditions (https://www.fondazioneslowfood.com/en/); (c) Ark of Taste: Slow Food online catalogue of the quality food products at risk of extinction (https://www.fondazioneslowfood.com/en/); (d) De.Co.: acronym for “*Denominazioni Comunali*” (i.e., “municipality designations”, known also as “De.C.O.”, acronym for “*Denominazioni Comunali d’Origine*”, i.e., “municipality designations of origin”), is a guarantee label assigned by Italian municipalities to food and agricultural products from their own territory, in compliance with Law 8 June 1990, n. 142.

## Distribution, Cultivation and Classification History

### A novelty in Europe: from the Torino Agricultural Garden to the first attempts at cultivation

Similarly to other maize landraces “discovered” during the early 20th century in Italy (e.g., ‘Mais reggiano’ or ‘Nano precoce’, see Succi, 1917), beaked maize was thought to have been introduced as a ‘Pisingallo’-like beaked popcorn from the Americas by a fabled emigrant farmer returning to his homeland (Zapparoli, 1926; Brandolini, 1955c). This hypothesis is likely a myth, since massive Italian emigration to the Americas started in 1861 (Del Boca & Venturini, 2003), when similar maize landraces were already known in Italy.

The earliest reference to a maize with beaked kernels in Europe dates back to Matthieu Bonafous (in Soulange Bodin, 1840), director of the Agricultural Garden in Torino (Ghisleni, 1996), who described under the name *Zea mays* (unranked) *rostrata* Bonaf. [“*zea maïs rostrata*”; the rank was specified later by Bonafous (1842a) as “*une simple variété*”, i.e., *Z. mays* var. *rostrata* (Bonaf.) Bonaf., see Art. 37.3 Ex. 4 of the ICN] a cultivated specimen of maize, present since 1837 in the aforementioned garden, not previously mentioned in his monumental monograph on this crop (Bonafous, 1836). It was described as having been provided with an early cycle as ‘Quarantino’ (or ‘Quarantin’, an early-cycle flint maize), more productive and “tasty” (with likely reference to its flour), and with a higher number of ears that were larger in size (Bonafous in Soulange Bodin, 1840; Bonafous, 1842a; Bonafous, 1842b). On the suggestion of the American botanist Robert Brown, he eventually tried to describe it as a new species (“*Zea rostrata*, *seminibus mucronatis*”, a phrase name, thus not to be regarded as a species name according to Art. 23.6(a) of the ICN; the correct name at species level was published by Poiteau (1842a) and Poiteau (1842b): *Zea mucronata* Poit.). In the same years, Metzger (1841) described a “*Rother spitzkörniger Mays*” (with dark-red and pointed to muticous kernels) obtained from the Berlin Botanical Garden, while Poiteau (1842a) reported the discovery, from some markets in France, of a similar maize with dark-yellow and pointed kernels, an earlier cycle and larger ears than ‘Quarantin’.

In Italy, as hoped by Bonafous (in Soulange Bodin, 1840), beaked maize started to spread as a novelty among agronomists and landowners, distributed by botanical/agricultural gardens and Bonafous himself (Bertoloni, 1845; Sanseverino, 1846). In 1843, it was reported as already present in Lombardia under the name of “*formentone rostrato di Lombardia*” (i.e., “beaked corn of Lombardia”) (Bizio, 1843), subsequently reaching the surroundings of Bologna (Bertoloni, 1845), probably Tuscany (Sanseverino, 1846), the provinces of Piacenza (Bracciforti, 1877) and Belluno (Bazolle, 1987), mostly for experimental cultivation purposes (Fig. 1). Sturtevant (1899) included Bonafous’ *Z. mays* var. *rostrata* within the popcorns (*Z. everta* Sturtev.), probably on the basis of its morphological affinity, communicated by Brown to Bonafous (1842a), with some ancient Peruvian stone ear-replicas of beaked popcorn (defined as “Proto-Confite Puntiagudo” by Grobman, Salhuana & Sevilla, 1961; see also Brown, 1934). Although the available descriptions of this early Italian beaked maize are sketchy, they evidence some elements, such as the appreciated quality of its flour and the remarkable productivity (Bertoloni, 1845; Bonafous, 1842a; Bonafous, 1842b) that clearly rule out Sturtevant’s classification, although some characteristics (e.g., multiple ears per plant, small kernels; see Bonafous in Soulange Bodin, 1840; Bertoloni, 1845) were still reminiscent of popcorn. Since accounts of accidental crossing episodes with other types of maize were already frequent (e.g., Bertoloni, 1845; Il Tornaconto, 1847; Lambruschini, 1847), it can be assumed that around the 1840s–1850s a transformation towards the current beaked flint/semi-flint form was taking place. Berti-Pichat (1863), in fact reported, under the names “*Formentone uncinato*, *a becco*” (i.e., “hooked corn, with beak”) and “*Zea m. rostrata*”, plants taller (>2 m) and with a longer maturing cycle than previous descriptions. Similarly, two illustrations of the “*Maïs jaune à bec*” published in France by Heuzé (1873), the first visual evidence of a European beaked maize, depicts an ear and kernels proportionally larger than typical popcorns and much more reminiscent of the current beaked representatives; yet, the relationship of this specimen with the Italian plants is unknown, although the description is clearly based on those provided by Bonafous.

### Successful spreading across the Po Plain

In 1914, a beaked maize cultivated by an agricultural cooperative (“*Probi contadini*”) in Comun Nuovo (province of Bergamo, see also Fig. 1), was presented, under the name of “*Rostrato*”, at a “corn farmers’ contest” in Bergamo. The plant, accurately described and photographed, was appreciated by the jury for the quality of its kernels and started to draw the attention of agronomists (Venino, 1916). Thanks to its productivity, in the mid-1920s ‘Rostrato’ started to be spread via agricultural institutions and private citizens from Comun Nuovo and nearby municipalities across most of the plain on the northern bank of the River Po. Between 1926 and 1936 it reached its widest range of distribution, with a remarkable diffusion in the provinces of Cuneo, Novara (where it occupied 15–20% of the area under maize, equal to circa 1,507–2,010 ha, see Istituto Centrale di Statistica del Regno d’Italia, 1936), Torino, Bergamo, Brescia, Pavia and the whole Veneto region (Brandolini, 1955a; Brandolini, 1955b; Zapparoli, 1926). Since at least 1932 it was additionally cultivated in Africa Orientale Italiana (A.O.I.), especially by the “Società Italiana Agricola Italo-Somala” (S.A.I.S.) in Italian Somaliland (Ciferri, 1942); probably in this period it also arrived in north-western Yugoslavia, now Slovenia and Croatia (Leng, Tavčar & Trifunović, 1962). Yet, in northern Italy ‘Rostrato’ did not dominate amongst maize cultivars and landraces, and was often cultivated near other types of corn. Due to its high irrigation requirements, abundant foliage and medium-long maturing cycle, it did not establish itself in the warmer areas on the southern bank of the River Po, where shorter-cycle and more drought-tolerant flint cultivars and landraces were preferred (Brandolini, 1955a; Brandolini, 1955b; Zapparoli, 1926).

According to Brandolini (1955a) and Zapparoli (1926), the plants from Comun Nuovo are commonly considered the direct precursors of the beaked maize now cultivated across northern Italy, based on a number of phenological and morphological characteristics close to the contemporary beaked landraces: late maturing cycle; tall stature, exceeding 2 m in height; wide leaves; ears subconical, usually one (rarely two) per plant; cob mainly white; kernels longer than 11 mm, beaked, with a paler spot on the crown; endosperm from flint to semi-flint. The combination of these characteristics proved to be key in distinguishing beaked maize in the context of the Italian maize germplasm classifications undertaken over the course of the 20th and the early 21st centuries that, after Succi (1931), definitively abandoned the employment of taxonomic ranks (such as species, subspecies and variety) in favour of other categories.

Considering the late maturity cycle, Zapparoli (1941) classified the beaked maize within the “*maggenghi*” (i.e., “May-time”) type, a category comprising cultivars and landraces that needed to be sown between the second half of April and the middle of May. Based on the examination of both morphological and phenological characteristics of the accessions acquired during the Italian maize germplasm collecting mission promoted in 1954 by the Stazione Sperimentale di Maiscoltura (now CREA-MAC) of Bergamo, Brandolini & Brandolini (2001), Brandolini & Brandolini (2006) and Brandolini & Brandolini (2009) grouped the beaked maize landraces into the “*Rostrato*” agroecotype, a member of the racial complex “Insubrian flints and semi-flints” or “*Padani*”.

### Decline

Although the subject of experimental mass selection, and inter- and intravarietal crosses for more than a decade, which resulted in the constitution of the formally improved yet unsuccessful cultivar ‘Rostrato Cajo Duilio’ and the Rostrato × Dentato Group (see Zapparoli, 1939a; Brandolini, 1955b; Brandolini & Brandolini, 2006), from about the mid-1930s ‘Rostrato’ suffered from increasing competition with the new generation of improved flint cultivars (e.g., ‘Nostrano dell’Isola Finardi’, ‘Marano’, ‘San Pancrazio’, ‘Barbina a 14 file di Tortona’). These cultivars, selected from local populations and subjected to formal improvement with the support of agronomic institutions, underwent significant diffusion between the 1930s and the Second World War, especially in the context of the autarchy promoted by the Italian Fascist regime. The cultivation of beaked maize was further affected by the first dent cultivars imported from the United States in the interwar period (Zapparoli, 1939b; Zapparoli, 1943; Parenti, 1944; Fenaroli, 1949; Brandolini, 1955b; Lanza, 1961; Brandolini & Brandolini, 2006), followed by the dent hybrids introduced from the same country: these were cultivated on a large scale from 1948 and in less than twenty years occupied more than 75% of the area under maize of the Po and Venetian Plains provinces, increasing to more than 90% in 1970 (Fenaroli, 1949; Fenaroli, 1955; Notiziario statistico, 1966; Notiziario statistico, 1973).

Already in 1954, the cultivation range of ‘Rostrato’ had become substantially contracted and fragmented. And, although it had completely disappeared from the Comun Nuovo territory, it survived in a few isolated localities of the Po and Venetian Plains (Cuneo, Novara, Trieste), and in some Alpine valleys (Trento, Bolzano, Belluno). In these areas, defined by Brandolini (1955b) as “secondary centers of cultivation”, in the course of the past two decades farmers had independently selected individual lines from the original ‘Rostrato’, provided with different characteristics from their ancestor, and better adapted to local climate conditions and agricultural needs (Brandolini, 1955a; Brandolini, 1955b).

### Today

Our field and literature surveys provided the first comprehensive distributional scenario of beaked maize in northern Italy after the 1954 national collecting campaign promoted by the Stazione Sperimentale di Maiscoltura of Bergamo (Brandolini & Brandolini, 2006).

**Figure 2:**
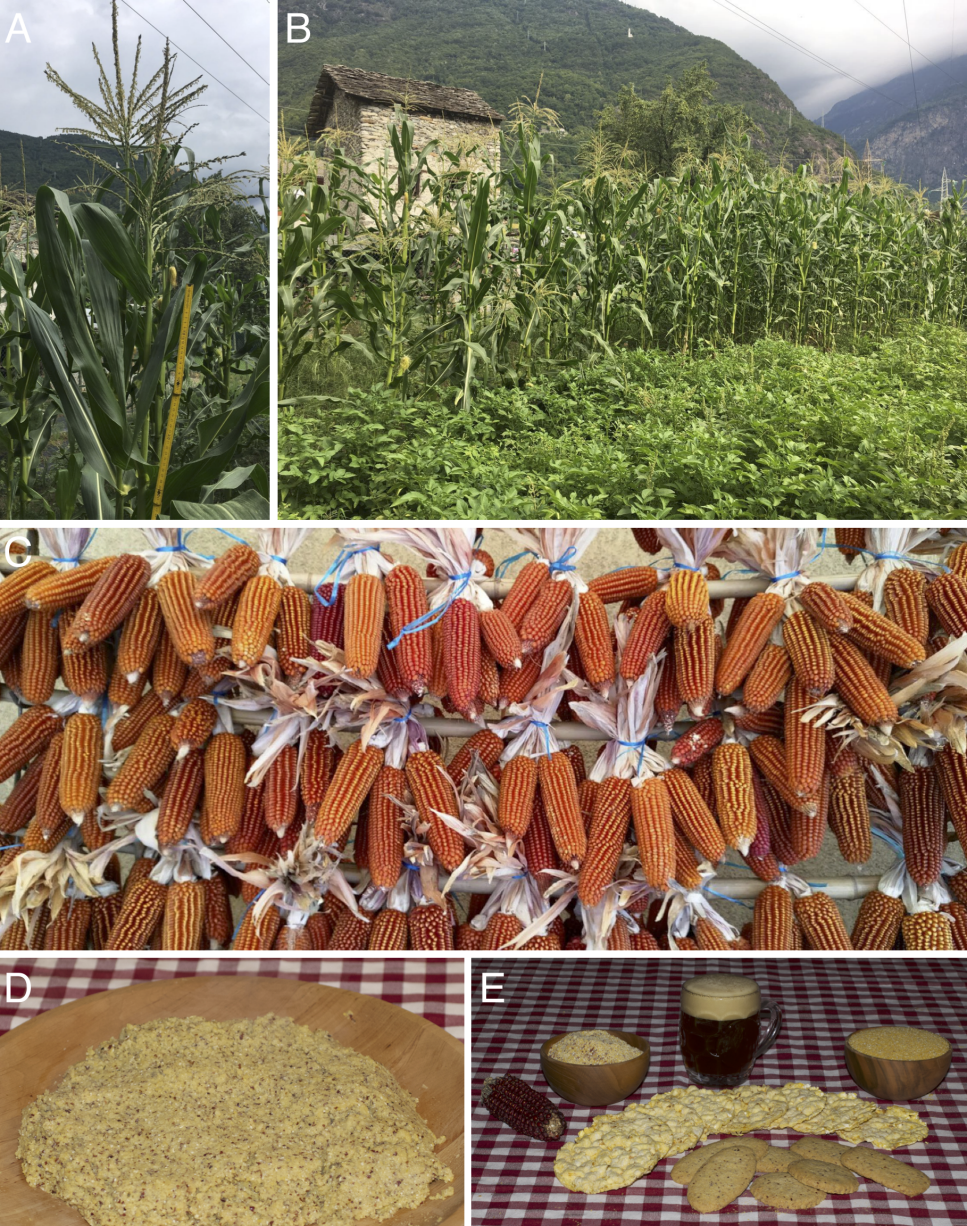
Cultivated plants, drying ears and products of *Zea mays* subsp. *mays* Rostrata Group. (A–B) Plants of ‘Rostrato di Valchiavenna’ cultivated in Chiavenna (Lombardia, Italy). (C) Ears of ‘Rostrato di Valchiavenna’ hung on bamboo canes for drying in Prata Camportaccio (Lombardia, Italy). (D) Polenta cooked with flour of ‘Rostrato di Mortara’. (E) Maize flour, beer, crackers and biscuits (made with ‘Rostrato di Mortara’, except the crackers and the flour on the right, obtained from ‘Dencìn della Valle del Ticino’). Photo credits: Graziano Rossi (A–C), Filippo Guzzon (D–E).

**Figure 3:**
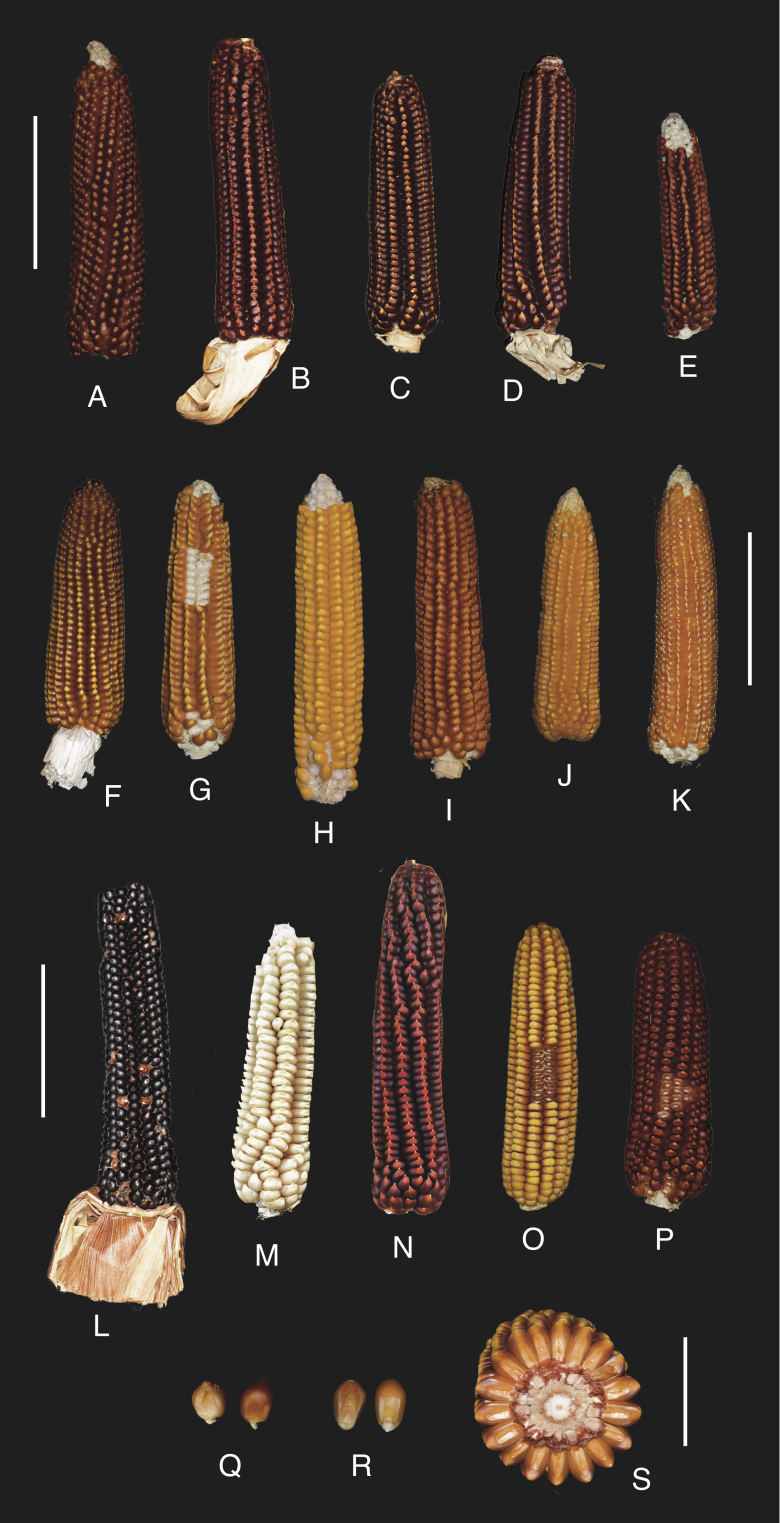
The Rostrata Group and crosses: representative ears, kernels and ear section. (A–P) Ears. (Q–R) Kernels. (S) Ear section. (A) ‘Spin di Caldonazzo’ (United States of America, as ‘Floriani Red Flint’). (B) ‘Rostrato di Mortara’ (Mortara, Italy). (C) ‘Rosso di Brescia’ (Pessina Cremonese, Italy). (D) ‘Rostrato rosso di Rovetta’ (Rovetta, Italy). (E) ‘Dencìn della Martesana’ (Inzago, Italy). (F, S) ‘Rostrato di Valchiavenna’ (Prata Camportaccio, Italy). (G) ‘Türc’ (Piateda, Italy). (H) ‘Spinato di Gandino’ (Gandino, Italy). (I) ‘Mais di Brumano’ (Brumano, Italy). (J) ‘Dencìn della Valle del Ticino’ (Robecchetto con Induno, Italy). (K) ‘Sponcio’ (Belluno, Italy). (L) ‘Nero spinoso’ (Esine, Italy). (M, N) ‘Nostrano di Pasiano’ (Pasiano di Pordenone, Italy). (O–P) ‘Rostrato di Valchiavenna’ with strong introgression of dent hybrid corn (Gordona, Italy). (Q) ‘Pignoletto della Val Cosa’ (Sequals, Italy). (R) ‘Spin di Caldonazzo’ (Trentino Seed Bank, Italy). Scale bar is 10 cm in A–P, and 3 cm in Q–S. Photo credits: Claudio Ballerini (A–L, O–S), Diego Pizzolato (M, N). Figure composition credit: Giulia Maria Francesca Ardenghi.

Currently, 23 different landraces of beaked maize are cultivated across the study area (R1, R2, R5, R7–R21, R23–R27) together with five crosses involving the genepool of these landraces (R3, R4, R6, R22, R28). No formally improved cultivars were identified. Eighteen landraces (R1–R4, R6, R8–R10, R12, R14, R16, R18, R22, R24–R28) are recorded for the first time in this publication as a result of the ethnobotanical investigation carried out by the authors (Table 1, Figs. 2 and 3).

The complex of landraces and allied crosses is distributed in six regions (Piemonte, Valle d’Aosta, Lombardia, Veneto, Trentino-Alto Adige, Friuli-Venezia Giulia) and 22 provinces of northern Italy; one (R20) is also cultivated in the United States (Table 1, Table S1, Fig. 1). Seventeen were identified in Lombardia, five in Friuli-Venezia Giulia, three in Piemonte and Veneto, one in Trentino-Alto Adige and Valle d’Aosta respectively; a single landrace (R6) is grown in multiple regions, i.e., Friuli-Venezia Giulia, Veneto and Lombardia. Most of the 83 localities of cultivation are scattered over the Po Plain (but only on the northern bank of the River Po), the Venetian Plain, Alpine valleys (ranging from the Western to the Eastern Alps) and hilly areas at the base of the Alps (Colline Novaresi, Colline del Varesotto, Collio); only three are located in the Subappennine and Northern Apennine (Fig. 1, Table S1). Eight landraces are cultivated only in plain areas (R1–R3, R9, R10, R12, R14, R16), 16 exclusively in mountain (R5, R17, R19–R28) or hill (R8, R13, R15, R18) localities, and the remaining four (R4, R6, R7, R11) occur both in plain and mountain or hill valleys (Table 1, Table S1). The total area under beaked maize in northern Italy can be estimated at around 150 ha, although data are not available for all the landraces and the surface devoted to their cultivation varies from year to year, due to, e.g., crop rotation and plant diseases. Landraces cultivated by single growers usually occupy less than 1 ha, such as R1 (circa 0.007 ha), R28 (0.07 ha), R25 and R26 (both circa 0.2 ha), R3 (circa 0.3 ha), while most of those grown by associations of farmers are extended over larger surfaces, such as R13 (4 ha), R19 (11 ha), R23 (circa 20 ha), R20 (circa 30 ha) and R21 (48 ha) (Ferrari, 2013; Arduin & Sanson, 2015; Buffoli, 2015; Gourmarte, 2016; Rottigni, 2017; http://www.antichimaispiemontesi.it/; http://www.pignolettorosso.it/; our interviews).

In continuity with the local tradition, all the landraces, being provided with flint/semi-flint endosperm, are employed in the production of maize flour used for cooking polenta. Its flavor differs among landraces, with, for instance, sweetish or bitterish variants (Zeppa & Rolle, 2003; our interviews). Maize flour is the main ingredient of several additional processed products: biscuits, pastries, cakes and crackers. Recently, new products trials have been undertaken, such as with beer, pasta, pizza and ice cream (Table 2, Fig. 2). With the exception of R3 and R6, characterized by a white endosperm, the flour of every landrace is yellow, with distinctive minute reddish to vinous-brown spots due to the pericarp residuals, which give polenta a peculiar grainy texture, quite similar to that of *polenta taragna* (made with both maize and buckwheat flours, see Riva, Nistri & Paolazzi, 2011).

**Table 2:** Products and their commercialization, germplasm origin, appraisal instruments and conservation measures associated with the investigated landraces. Each landrace is indicated by means of the codes employed in Table 1. In column “Prom./Prot.” (abbreviations for “promotion and protecting measures”), the acronym “NCVR” stands for the Italian National Conservation Varieties Register. In column “Genebanks”, the following abbreviations are used: “CREA-MAC”, Unità di Ricerca per la Maiscoltura of Bergamo; “Pavia”, University of Pavia Germplasm Bank; “Strampelli-Lonigo”, Istituto Strampelli of Lonigo germplasm bank; “Svalbard”, Svalbard Global Seed Vault; “Trentino”, Trentino Seed Bank of MUSE—Museo delle Scienze di Trento, Trento. Sources investigated for each landrace are reported in Table S1.

**Code**	**Product(s)**	**Commerce**	**Germplasm origin**	**Prom./Prot.**	**Active conservation**	**Genebanks**
R1	Maize flour	Yes	Recovered landrace	None	No.	Pavia
R2	Maize flour, crackers	Yes	Native landrace	None	In order to prevent crossing with dent hybrids, it is cultivated in small woodland clearings in the Ticino Valley (despite damages by wild boars), following the advices of the farmer who donated the seeds; in the past, he cultivated this landrace also in water meadows (*marcite*).	Pavia
R3	Maize flour	Yes	Native landrace	Association	Red kernels, not appreciated on culinary grounds (they produce a “greyish” flour), are instead traditionally sown to obtain plants with a remarkable pollen production, that is locally believed to prevent introgression from dent hybrids. It is usually cultivated a long distance away from dent hybrid fields.	Pavia
R4	Maize flour	No	Cross	None	Cultivated at a distance of 10 km from dent hybrid fields to prevent crossing.	Pavia
R5	Maize flour	Yes	Native landrace	NCVR	Cultivation was historically conducted in the isolated mountain locality of Annunciata, in order to prevent crossing with other types of maize.	CREA-MAC; Pavia
R6	Maize flour	No	Native landrace	None	No.	Pavia
R7	Maize flour, pastries (*paste di meliga*)	Yes	Native landrace	NCVR; association	Cultivated distance of at least 300 m from dent hybrid fields, often in fenced areas to prevent damage by wild animals.	CREA-MAC
R8	Maize flour	Yes	Native landrace	None	No, but aware of problematic dent hybrid introgression.	Pavia
R9	Maize flour	Yes	Introduced landrace	None	No, but aware of problematic dent hybrid introgression.	None
R10	Maize flour	Yes	Native landrace	None	Cultivated a long distance from the dent hybrid fields.	None
R11	Maize flour, crackers	Yes	Native landrace	NCVR; association	Cultivated at a distance of at least 300 m from dent hybrid fields, often in fenced areas to prevent damage by wild animals.	CREA-MAC; Strampelli-Lonigo
R12	Maize flour	Yes	Native landrace	Association	Cultivated a long distance from the dent hybrid fields.	None
R13	Maize flour, cakes	Yes	Native landrace	Association	Cultivated on a small piece of land (4 ha) surrounded by woodlands in order to prevent crossing with dent hybrids.	None
R14	Maize flour, biscuits	Yes	Introduced landrace	None	No.	Pavia
R15	Maize flour, cakes	No	Recovered landrace	None	Unknown.	CREA-MAC
R16	Maize flour, biscuits, beer	Yes	Recovered landrace	None	The sowing time is planned in order to prevent simultaneous flowering with dent hybrids; moreover, at harvesting time individuals located on the external fringe of the field are eliminated.	Pavia
R17	Maize flour, beer	No	Native landrace	None	Cultivated a long distance from other maize fields.	CREA-MAC; Pavia
R18	Maize flour, crackers	Yes	Native landrace	None	No, but this landrace is being informally improved.	CREA-MAC
R19	Maize flour, biscuits, cakes, crackers, ice cream	Yes	Native landrace	NCVR; Ark of Taste; De.Co.; association	G Marinoni maintained this landrace by collecting the ears manually, preventing that seeds mixed with those of other maize cultivars/landraces in combine harvesters. The association “Rosso Mais” supervises the choice of the fields, in order to prevent any crossing with other types of maize.	CREA-MAC; Pavia; Svalbard
R20	Maize flour	Yes	Native landrace	Ark of Taste; De.Co.	In compliance with the regulation for the production of maize flour under the label “Qualità Trentino”, fields need to be at a distance of at least 300 m from those of other maize cultivars/landraces. Only ears from plants with features typical to this landrace are selected for the seeds production.	CREA-MAC; Pavia; Trentino
R21	Maize flour, crackers, bread, stuffed pasta, pizza, biscuits, cakes, ice cream, beer	Yes	Recovered landrace	NCVR; Ark of Taste; De.Co.; association	In compliance with De.Co regulation, fields need to be isolated, at a distance not less than 200 m from other maize cultivations, that have to be reported by the “custodian farmers” to the De.Co commission (natural and urban barriers are not regarded as protective). Plants not typical need to be eliminated before the flowering of tassels.	CREA-MAC; Pavia; Svalbard
R22	Maize flour, biscuits	Yes	Cross	None	No.	None
R23	Maize flour, pre-cooked polenta, pasta, crackers, biscuits	Yes	Native landrace	None	Yes, but details unknown.	Pavia; Strampelli-Lonigo
R24	Maize flour	No	Native landrace	None	Unknown.	Pavia
R25	Maize flour, crackers	Yes	Introduced landrace	None	Cultivated at a long distance from the dent hybrids fields; when this is not possible, sowing time is brought forward by about 20 days.	None
R26	Maize flour, crackers	Yes	Introduced landrace	None	Same as R25.	None
R27	Maize flour	No	Native landrace	None	Unknown.	None
R28	Maize flour	No	Cross	None	Cultivated at a distance of more than 350 m from other maize fields. No chemicals are employed and the yield is sometimes damaged by Eurasian magpies and Eurasian badgers.	Pavia

Recently, outside northern Italy, three accessions of beaked maize with flint to semi-flint kernels have also been sampled in south-eastern and south-western France (Pro-Maïs, 2018).

## Identity of *Zea Mays* Subsp. *Mays* Rostrata Group

### Classification and description

Zapparoli (1926), Brandolini (1955c), Brandolini (1970), Brandolini & Mariani (1968) and Brandolini & Brandolini (2006) assumed that the current assemblage of beaked maize landraces with yellow and flint/semi-flint endosperm probably originated in Italy as a result of natural and/or informal (i.e., unconscious or directed by individual farmers and growers; see Cleveland, Soleri & Smith, 2000; Camacho Villa et al., 2006) crossing among three types of maize introduced from the Americas, based on a peculiar combination of morphological traits: (1) the presence of beaked kernels and prominent rows can be referred to a descendant of pointed/beaked popcorn from Central and South America (e.g., ‘Canguil’, ‘Confite Puntiagudo’, ‘Imbricado’, ‘Pisankalla’ and ‘Pisingallo’ or ‘Pisincho’; see Roberts et al., 1957; Brieger et al., 1958; Ramírez et al., 1960; Grobman, Salhuana & Sevilla, 1961; Timothy, Peña & Ramírez, 1961; Solari & Gómez, 1997; Santacruz-Varela et al., 2004); (2) additional kernel (dominant flint endosperm and large size), ears (exceptional length) and vegetative (abundant foliage and tall stature) characteristics were likely inherited from local flint landraces, like ‘Pignoletto’ (or ‘Pignolo’) and ‘Agostano’ (see Brandolini & Brandolini, 2006); (3) the not infrequent occurrence of indentation and a small percentage of soft endosperm indicates the involvement of ancestral dent landraces, most likely descendants of ‘Shoepeg’ from the southern United States (see Brown & Anderson, 1948).

Taking into consideration the historical account of beaked maize in Europe given in the previous paragraph, three of these morphological characteristics constantly appear in all the available descriptions published since Bonafous (in Soulange Bodin, 1840; Bonafous, 1842a; Bonafous, 1842b) and characterize every landrace with yellow endosperm to emerge during our ethnobotanical surveys: (1) kernels with a distinct apical beak; (2) flint endosperm (employed to produce flour for polenta); (3) ears of exceptional length. Since the second half of the 19th century, as a consequence of the afore-documented continual introgression, beaked maize cultivated in Italy started to acquire some traits not reported or divergent from those originally described by Bonafous: (1) maturing cycle longer; (2) stature taller (exceeding 2 m); (3) well-developed foliage; (4) reduction of the number of ears per plant (from multiple to one). The complex of these seven “ancestral” and “recent” phenological and morphological traits is shared by all the landraces of beaked maize with yellow endosperm cultivated in Italy since the second half of the 19th century. Based on this similarity, these landraces can be designated as a Group, in compliance with Arts. 3.1 and 3.2 of the ICNCP. Particularly, according to Arts. 3.3 and 22 of the ICNCP, the correct name to be adopted is Rostrata Group, being based on *Zea mays* (unranked) *rostrata* Bonaf., the earliest available name under the ICN (see also the previous paragraph).

On the basis of literature (Arduin & Sanson, 2015; Bertolini, 2002; Bertolini, Franchi & Frisanco, 2005; Brandolini, 1955a; Brandolini & Brandolini, 2001; Brandolini & Brandolini, 2006; Buffoli, 2015; CRAB, 2004; Gruppo di lavoro cerealicolo, 2014; Veneto Agricoltura, 2014; Regione Lombardia, 2017; Venino, 1916; Zapparoli, 1926; http://www.antichimaispiemontesi.it/) and original measurements of the accessions stored at the University of Pavia Germplasm Bank (see Table 2), a description of the Rostrata Group is here reported with the aim of summarizing its variability and updating previous descriptive accounts (such as Brandolini & Brandolini, 2006) not taking into consideration the landraces uncovered in the last few decades: *maturing cycle* variable, but mostly medium-late to late. *Plants* robust and tall, reaching up to 2.5 m or more in height; tillers absent; leaves abundant, leaf blades wide, usually deep green, occasionally purplish-brown. *Staminate inflorescences* (“tassels”) more than 40 cm long, highly branched. *Pistillate inflorescences* (“ears”) usually one (rarely two) per plant, located at about the middle of the culm; prophyll and leaf sheaths (“husks”) deep green, sometimes purplish-brown. *Catoclesia* (“ears”) 12–25 cm long, 3.5–5.5 cm wide, cylindro-conical to cylindrical, base slightly enlarged or not, apex rounded to acute; rows 12–18, usually regularly arranged and straight, from widely spaced to packed, resulting in a star-shaped to round ear cross-section respectively; rachis (“cob”) with white or pinkish to dark brownish-purple (“red”) glumes. *Caryopses* (“kernels”) 8–13 mm long, 7–10 mm wide, 2–5 mm thick, broadly elliptical to broadly obovate, apex from roundish to pointed arch-shaped, usually tapering, in correspondence of the style insertion, into a prominent and prickly beak curved distalwards, sometimes reduced only to the enlarged stylar base; crown smooth or with a shallow to deep indentation; pericarp from bright yellow to deep orange or from red to chocolate- or vinous-brown (“black”), concolor or with a paler (usually yellow to orange-yellow) spot of variable extension on the crown due to the presence of floury (“soft”) endosperm; endosperm entirely horny (“flint”) or with a small proportion of floury endosperm (“semi-flint”), yellow (Figs. 2, 3A–3H, 3J–3K and 3Q–3S).

As observed by Zapparoli (1926), the Rostrata Group possesses an intrinsic morphological and phenological variability that is associated with its heterogeneous origin. Being open-pollinated, its variability can be further emphasized as a result of spontaneous crossings with other types of maize cultivated nearby: thus, as evidenced by experimental crossings by Brandolini (1955c), under the influence of modern dent hybrids, cob turns red, floury endosperm and kernel indentation increases, pericarp color becomes yellower and/or beak disappears (see also Figs. 3O–3P); on the other hand, flint cultivars (like ‘Nostrano dell’Isola’ and ‘San Pancrazio’) and landraces can promote a reduction of row prominence, and kernel and beak length.

### Similar groups

The Rostrata Group should not be confused with other assemblages of landraces occurring in the study area, similarly provided with beaked kernels but distinguished for other traits such as the endosperm type and their presumed different origin.

It is the case of the Rostrato bianco Group, distributed mainly in north-eastern Italy: classified by Brandolini & Brandolini (2001), Brandolini & Brandolini (2006) and Brandolini & Brandolini (2009) as an agroecotype within the racial complex “White Dents”, it encompasses landraces similar in most phenological and morphological characteristics to the Rostrata Group (i.e., very late maturing cycle, well-developed foliage, beaked and long kernels), except that kernels are constantly indented, slightly wider, with ivory-white pericarp and white and floury endosperm. Its origin is different, being a likely derivative of Mexican ‘Pepitilla’ (Wellhausen, Fuentes & Hernandéz Corzo, 1957; Brandolini & Mariani, 1968; Brandolini, 1970).

On the other hand, the Rostrato × Dentato Group, which includes crosses between the Rostrata Group landraces with white dents (probably descendants of ‘Shoepeg’) introduced before modern dent hybrids (Brandolini, 1955b; Brandolini, 1970; Brandolini & Brandolini, 2006), is characterized by taller stature (>3 m) and wider, broadly elliptical to almost isodiametric and deeply indented kernels, provided with white endosperm (Figs. 3M–3N). It was obtained in the 1930s by the Centro provinciale di maiscoltura of Udine mainly for forage purposes but was also appreciated for the grain quality (Zapparoli, 1938; Zapparoli & Parenti, 1943; Consorzio Agrario provinciale Udine, 1950; Brandolini & Brandolini, 2006).

## Back to Beaked: Present State of Conservation and Revival

### Germplasm origin

In order to understand the distribution patterns across time of the current landraces and allied crosses of beaked maize, we arranged them into four different categories (Table 2). These were fundamental to define, identify and locate refugia and the revival process (see the following paragraphs) and are presented here:

(1) *Native landraces*: cultivated in the same area by, at least, two generations of farmers; we also included within this category landraces whose cultivation has recently been expanded outside of their native range.
(2) *Recovered landraces*: re-propagated from kernels stored *ex situ*, either in crop genebanks or otherwise (e.g., ears hung on buildings’ walls).
(3) *Introduced landraces*: cultivation derived from germplasm imported from a different area than the one in which they are currently cultivated; contrary to *native landraces* that have extended their previous range, *introduced landraces* cannot be found anymore in their native area from where they were imported.
(4) *Crosses*: resulted from a recent deliberate cross between two landraces. Crosses can be further divided into *native* and *introduced crosses*. In the first case, the parental landraces are both native to the area where the cross originated; in the second case, at least one of the parental landraces has been introduced from a different area to where the cross originated.

As shown in Table 2, native landraces are 17 (R2, R3, R5–R8, R10–R13, R17–R20, R23, R24, R27), including the two landraces belonging to the Rostrato × Dentato Group (R3, R6); recovered landraces are four (R1, R15, R16, R21), the same number as the introduced landraces (R9, R14, R25, R26), while crosses are three (R4, R22, R28). This latter category comprises only introduced crosses, originated between the 1980s and the 2000s: the parental landraces of R4 and R28 were imported from different areas from the one where these crosses originated and are now cultivated, while R22 resulted from the cross of a local landrace and an imported one; all the crosses are cultivated only in the municipality where they originated.

It is interesting to note that all three crosses are the result of informal crossing between a beaked and a non-beaked flint landrace cultivated in contiguity, then deliberately maintained by individual farmers for specific traits of culinary and aesthetic interest. Similarly, as for the landrace R16, the farmer who originally recovered it is selecting a new line characterized by black and beaked kernels. One of the farmers who is cultivating R5 is likewise selecting a new line provided with red kernels (Table 1). These observations, inserted into the debate about the most suitable definition of a landrace, highlight the fact that landraces, even in an intensive cropping system, are dynamic entities far from being merely relic entities doomed to extinction. Instead, they are constantly subjected to evolutionary forces, in terms of local environmental conditions, farmer selection and crossing with other landraces (Zeven, 1998; Camacho Villa et al., 2006; Casañas et al., 2017).

These considerations are supported by the origin of most OPVs and specifically the Rostrata Group itself, which arose from the informal crossing of different maize types that were cultivated for a prolonged period in close proximity (“*vicinismo*”, i.e., “vicinity”, as defined by Zapparoli, 1926). These promoted a dynamic “exchange” of characteristics that proved to be decisive in the process of adaptation to local environmental conditions and in satisfying specific food or agricultural needs (Brandolini & Brandolini, 2006).

### Conservation

As proposed by Lanza (1961), maize cultivation history between the 19th and 20th centuries in Italy can be divided into three major periods, each one represented by a different generation of maize cultivars and/or landraces that almost completely replaced its predecessor. The Rostrata Group belongs to the first of these generations, which included local OPVs not subjected to formal improvement, pre-dating the generations of the 1920s–1930s improved cultivars and the United States dent hybrids imported into Italy since the late 1940s (see also the “History” subheading). Given that, the genepool of the Rostrata Group, not contributing to extant improved lines (‘Rostrato Cajo Duilio’ remained confined to the experimental context), may contain untapped allelic variation useful for future breeding programmes and therefore should be given a priority ranking in germplasm conservation (Reif et al., 2005), similar to other “first generation” maize landraces still available in northern Italy, such as ‘Badoera’, ‘Caragua’, ‘Pignoletto d’oro’ and ‘Taiolone’ (Zapparoli, 1941; Consorzio Agrario provinciale Udine, 1950; Lanza, 1961; Brandolini & Brandolini, 2006; Riscoperto il Pignoletto d’oro, mais del ’700: pasticceri pronti alle ricette, 2015).

Given the importance of beaked maize germplasm conservation, we provided a further insight into its on-farm conservation by identifying measures put in place by farmers to prevent introgression events with hybrid dent corn. The awareness of farmers about the threat represented by the introgression of hybrid dent cultivars in the maintenance of OPVs morphologically and genetically true-to-type was identified for 20 landraces (R2–R5, R7–R13, R16, R17, R19–R21, R23, R25, R26, R28). While for 18 landraces active conservation measures are put in place (R2–R5, R7, R10–R13, R16, R17, R19–R21, R23, R25, R26, R28), for two landraces (R7, R8) the interviewed farmers wished to be supervised by an agronomic institution to help reduce introgression events. For only four (R13, R19–R21) of the 18 landraces in which active conservation measures were observed, those measures follow procedural guidelines prescribed by farmers’ associations or De.Co (Table 2).

The main on-farm conservation strategies to prevent introgression issues are:

(1) sowing a long distance, of at least 200 m, from other maize cultivations, especially of hybrid dent corn (R3, R4, R7, R10–R12, R17, R19–R21, R25, R26, R28). In some cases (i.e., R2, R5, R13), isolated sites, such as clearings within woodlands or poorly accessible mountain fields, are preferred for the cultivation of beaked maize, in spite of the risk of possible damage from wild animals (e.g., wild boars) and thus yield loss;

(2) sowing in different periods from hybrid dent maize to avoid simultaneous flowering (R16);

(3) selecting, through mass selection, ears and kernels with the characteristic features of the landrace (e.g., R20, R21; Table 2).

Even if guidelines for the on-farm conservation, and in particular the avoidance of introgression by hybrid dent corn, are available for only four landraces (R13, R19–R21), for 20 landraces farmers are aware of and/or have directly experienced the issue of dent hybrid introgression, with 18 of them carrying out active measures to prevent it or at least minimize it (Table 2). The *in situ*, i.e., on-farm conservation measures can therefore be considered adequate even if not guided, in most cases, by any scientific institution dealing with agrobiodiversity conservation.

The *ex situ* conservation status of the Rostrata Group genepool appears satisfactory: for 20 out of the 28 landraces seed samples are maintained in long-term storage in one or more seedbanks (R1, R2, R4, R5, R7, R8, R11, R14–R17, R19–R21, R23, R24). Of these, seven were conserved *ex situ* for the first time under the framework of the current study (Table 2).

### Refugia

As evidenced by our historical investigation and similarly to most OPVs in Europe, the progressive evolution of the maize cultivation scenario, especially after the introduction of hybrid dent corn, led to a drastic shrink in the cultivation of beaked maize, which, since the early 1950s, survived only in a few localities (see the “History” subheading). Nowadays, Rostrata Group landraces are still grown across most northern Italian regions, yet this extended distributional area is much more fragmented than it was 60 years ago (Fig. 1). Nevertheless, as far as could be ascertained from our ethnobotanical interviews and literature sources, the cultivation of more than half of these landraces (i.e., the 17 native landraces, see Table 2) never ceased completely over the course of the last 80 years in localities that can be regarded as “refugia” (Table 1, Table S1). Disentangling the reasons that allowed the persistence of these landraces in their refugia, two main factors emerged as key in their maintenance and survival: (1) the presence of physical barriers that prevented crossing with hybrid dent cultivars and (2) deliberate on-farm conservation performed by farmers.

Undoubtedly, cultivation in hill and mountain areas provided natural barriers that limited introgression events, first of all the distance from the Po and Venetian Plains, which host the most widespread cultivation of dent hybrid corn. Mountain areas are less suitable to intensive maize cultivation and the replacement of OPVs by hybrid dent cultivars has historically been slower in mountain provinces than in provinces located in the plains (Anonym, 1975; CRAB, 2004; I.Stat, 2018). Some landraces certainly profited of these conditions, such as ‘Nero Spinoso’ (whose cultivation has been preserved for about a century thanks to its circumscription to a remote mountain area, see Tables 1 and 2), leading researchers to hypothesize that isolated mountain sites are preferential for the maintenance of Italian OPVs (Bitocchi et al., 2009; Brandolini & Brandolini, 2009). Yet, by examining the geographical distribution of the 17 native landraces of beaked maize that were identified, only seven of these are exclusive to mountain territories, while ten are solely or mainly cultivated in plain or hill areas. Within this second group, four (R2, R3, R10, R12) are located in the middle of the Po and Venetian Plains and a fifth (R6) has expanded only recently to a single mountain locality from its plain area of origin (Tables 1 and 2). The preservation of these five landraces, whose native ranges have been rapidly and extensively colonized by dent hybrid corn, was only possible by means of active conservation measures personally developed by farmers: R2 is traditionally cultivated in clearings and water meadows within woodlands located below terraces of the River Ticino, while fields of R10 and R12 are commonly positioned a long distance from dent hybrids. It can be assumed that the persistence in Friuli-Venezia Giulia of R3 and R6, both belonging to the Rostrato × Dentato Group, was additionally helped by the strong traditional predilection, almost exclusive of the Venetian territories, towards white polenta over the yellow (Brandolini & Brandolini, 2006). In light of the relatively high number of native landraces that have their refugia not exclusively in mountain areas (10 out of 17) and the survival of five of them in plain areas, it is possible to reject the “mountain refugia hypothesis” and the paradigm that landraces survived genetic erosion simply because they were confined to isolated areas (Hawkes, 1983; Al Khanjari et al., 2007; Kasiotis et al., 2009). Moreover, these observations demonstrate that, in the absence of substantial natural barriers, deliberate on-farm conservation measures play a major role in establishing solid and permanent refugia for OPVs, highlighting the decisive role of “custodian farmers” (Gruberg et al., 2013; Sthapit, Lamers & Rao, 2013) and the consumption of traditional food products in preventing genetic erosion and safeguarding agrobiodiversity (Mendes Moreira & Veloso, 2009; Galluzzi, Eyzaguirre & Negri, 2010; Casañas et al., 2017).

### Revival

From our investigation it emerged that, after decades of decline, a revival process (in terms of an increased number of cultivation localities, product commercialization and promotion measures) involving beaked maize landraces has been occurring since the late 1990s and indeed has been gaining momentum since the early 2000s.

Of the 28 landraces investigated, the cultivation of four (R1, R15, R16, R21), whose cultivation ceased in the past (i.e., “recovered landraces”), has been re-established since the early 2000s, thanks to seed acquired from agronomic genebanks (e.g., CREA-MAC of Bergamo) or from old ears casually discovered in farms (Table 1). This is the single most important aspect of the revival on conservation grounds. Since the late 1990s, 10 (R2, R5, R7, R8, R11–R13, R19, R20, R23) out of the 17 landraces whose cultivation never ceased (i.e., “native landraces”), profited from a growing interest in their conservation, which resulted in an increased number of cultivation localities, such as R19, originally limited to Rovetta, and currently cultivated in other nearby municipalities. Some landraces went far beyond the boundaries of their native distribution range: it is the case of R7 and R11, native to the Canavese subalpine area and now cultivated across most of the Piemonte Po Plain and even in the Subapennine and Northern Apennine (where no historical evidence of their presence is available); or R6 from Friuli-Venezia Giulia which reached Marano Vicentino in Veneto and Songavazzo in Lombardia, where farmers, although already in possession of their own valuable cultivars and landraces (‘Marano’ and ‘Rostrato rosso di Rovetta’ respectively), are motivated to experiment with new crops simply out of curiosity or because traditional needs are not satisfied by local landraces (i.e., the white flour demand from the Lake Iseo area, see Table 1). For similar reasons, some landraces have also been introduced to new areas of cultivation (i.e., R9, R14) (Table 1). Despite the fact that in these instances the more or less informal exchange of seeds has crossed municipal, provincial and regional boundaries, a strong sense of territorial identity is felt: as revealed by our interviews, many farmers regard their “own” landrace as superior, for instance in the quality of flour, compared with those cultivated in neighbouring provinces or even within the same region (see e.g., Gandino, 2015). Although the number of localities increased in the last two decades, the current surface under beaked maize in northern Italy, equal to circa 150 ha, is much smaller than that of the interwar period, when beaked maize covered circa 1,500–2,000 ha only in the province of Novara (see the “History” subheading).

The revival of beaked maize is indissolubly linked to an increasing interest by consumers in traditional food products and their higher sensorial value, similarly to what is happening to other OPVs in Europe (see e.g., Lucchin, Barcaccia & Parrini, 2003; Lago et al., 2015). This phenomenon is highlighted by the fact that only seven landraces (R4, R6, R15, R17, R24, R27, R28) are cultivated by farmers solely for self-consumption. On the other hand, the products of the remaining 21 (R1–R3, R5, R7–R14, R16, R18–R23, R25, R26) are being commercialized (Table 2), usually sold by farms directly to the public (sometimes with the aid of a website or a social media page) or, in some cases, to restaurants, mills and grocery stores; participation in local festivals often represents an occasion for widening the clientele (Slow Food Provincia di Varese, 2017; http://www.pignolettorosso.it/; our interviews). We are therefore witnessing a shift in beaked maize cultivation that until the late 1990s was mostly designated for own-consumption, while currently it is mainly intended for commercialization. These landraces have survived genetic erosion as heirloom varieties and are now offering new economic opportunities to farmers. In this context, it is interesting to note that besides traditional products made using beaked maize flour, such as polenta and biscuits, new food products for some landraces (R2, R11, R16, R17–R19, R21, R23, R25, R26) are now being experimented with, such as beer, crackers and even pizza or ice cream (Table 2, Fig. 2). Similarly, a change in the utilization of two landraces (R3, R6) has been recorded: they belong to the Rostrato × Dentato Group that was originally obtained mainly for forage purposes (Consorzio Agrario provinciale Udine, 1950) before being replaced by dent hybrid corn, while they are now used for the production of white flour (Table 2).

Revival seems mostly spontaneously driven by farmers and consumers and only partially mediated by governmental and research institutions. It is emblematic that from 2010 to 2016 only five landraces (R5, R7, R11, R19, R21) have been registered in the National Register of Conservation Varieties and only three (R19–R21, two of which are listed in the aforementioned register) have been granted a De.Co. (for R21 the De.Co. label is applied not to the landrace itself but to *Farina di Melgotto*, the flour produced from this landrace) and Ark of Taste labels, while not one is listed as a Slow Food Presidium. It appears clear that, besides a few widely recognized landraces, there are several others which are undergoing a revival process via non-governmental means, such as associations of local farmers or millers, constituted around seven landraces (R3, R7, R11–R13, R19, R21) with the aim of promoting products derived from these and to safeguard the true-to-type cultivation (Table 2).

## Conclusions

Taking into consideration maize cultivation history in Italy from the 19th century up to the present day, marked by three generations of cultivars and landraces (Lanza, 1961), we can safely state that this country is now experiencing the rise of a fourth maize generation. It is made up of landraces that have been recovered thanks to the presence of refugia and a revival phenomenon after seven decades of genetic erosion. Coexisting and not competing with the current assemblage of modern cultivars dominated by hybrid dent corn (beaked maize cultivation accounts for only circa 0.02% of the total maize cultivation area in northern Italy), it occupies a different and well-defined cultural and economic niche, built around the rediscovery of traditional foods and agriculture promoted by a growing synergy between farmers and consumers (Bertolini, Franchi & Frisanco, 2005; Riscoperto il Pignoletto d’oro, mais del ’700: pasticceri pronti alle ricette, 2015; Lago et al., 2015; Femia, 2017; http://www.antichimaispiemontesi.it/). The Rostrata Group represents one of the most ancient and neglected genepools among this new crop generation, its origin chronologically preceding the improved cultivars of the 1920s and 1930s. The process of revival is contributing to its preservation and, to some extent, is providing an additional income to farmers.

In this context, the activities of local farmers, in the role of “custodians” of plant genetic resources need to be encouraged and supported, especially by government (in the framework of Italian Law 1 December 2015, n. 194) and research institutions. In relation to OPVs, custodian farmers appear in fact to be key figures in the accomplishment of on-farm conservation, whose role is eminent in promoting a dynamic adaptation of landraces to environmental and agricultural changes that, for instance, are being experienced in recent years by maize cultivation across Europe (Assosementi, 2016; Lesk, Rowhani & Ramankutty, 2016; Osservatorio Agroalimentare, 2017; Aoi, 2017; Iotti, 2017).

The first record in this paper of 18 beaked maize landraces previously unknown to the scientific community, also highlights the potential of ethnobotanical surveys in discovering neglected genetic resources (see, e.g., Gao, 2003; Shewayrga & Sopade, 2011) in areas characterized by industrial agriculture and a high degree of genetic erosion, as already evidenced by Ardenghi et al. (2017). These landraces, having not been preserved in any institutional genebank until this research, owe their survival only to the initiative of private farmers. In the light of this, further field research is urgently needed across Europe countries into similar groups of “first generation” maize landraces (such as white flints and dents or popcorns, see Brandolini & Brandolini, 2006), whose abandonment may lead to their extinction and therefore to the loss of untapped genetic resources.

##  Supplemental Information

10.7717/peerj.5123/supp-1 Data S1 Ethnobotanical interview form Ethnobotanical interview form used in surveys.

10.7717/peerj.5123/supp-2 Table S1 Municipalities where each cultivar is cultivated and associated investigated sources

## Additional Information and Declarations

### Data Availability

The following information was supplied regarding data availability: The raw data/code is included in the article (Tables 1 and 2; Table S1).
