# Supplementary material for: Back to beaked: Zea mays subsp. mays Rostrata Group in northern Italy, refugia and revival of open-pollinated maize landraces in an intensive cropping system
Source: PeerJ. 2018 Jul 4;6:e5123. doi: 10.7717/peerj.5123 (PMC6035727; doi:10.7717/peerj.5123)
Supplement: Data S1 — Ethnobotanical interview form used in surveys. [file peerj-06-5123-s001.docx]

**Ethnobotanical interview form**

(note: the current document is a translation of the Italian original)

Date, place and author(s) of the interview

**1. Personal information about the grower**

1.1. Name and surname

1.2. Date and municipality of birth

1.3. Municipality of residence

**2. Information about the maize landrace**

2.1. Local name of the landrace

2.2. Where do you cultivate it?

2.3. Since when are you cultivating it?

2.4. From where and how did you obtain the germplasm?

2.5. Is there any other growers that you are aware of, that cultivate this landrace?

2.6. Does this landrace have any peculiar feature that allow to distinguish it from others?

2.7. For which purpose do you cultivate it? Do you obtain any food product from it? Do you commercialize any of them?

2.8. Did you give seeds of this landrace to other farmers?

2.9. Do you know any similar maize landrace or cultivar?

2.10. Do you have historical information about this landrace (e.g., since when it is cultivated and its cultivation range in the past)?

**3. Information about the cultivation of the cultivar**

3.1. Time of sowing and yielding

3.2. Are you aware of introgression issues with hybrid dent corn?

3.3. If yes, do you put in place any measure to limit it?

**4. Additional notes**
