# Supplementary material for: Back to beaked: Zea mays subsp. mays Rostrata Group in northern Italy, refugia and revival of open-pollinated maize landraces in an intensive cropping system
Source: PeerJ. 2018 Jul 4;6:e5123. doi: 10.7717/peerj.5123 (PMC6035727; doi:10.7717/peerj.5123)
Supplement: Table S1 [file peerj-06-5123-s002.docx]

**Municipalities where each landrace is cultivated and associated investigated sources.**

| **Code** | **Landrace name** | **Distribution [*province(s)*: municipalitie(s)]** | **Region(s)** | **Sources** |
| --- | --- | --- | --- | --- |
|  |  |  |  |  |
| R1 | Dencìn [della Martesana] | *Milano*: Inzago, Gorgonzola | Lombardia | Our interview |
| R2 | Dencìn or Scagliolo [della Valle del Ticino] | *Milano*: Cassinetta di Lugagnano, Robecchetto con Induno | Lombardia | Our interviews |
| R3 | Dente di cavallo [del Friuli Orientale] | *Gorizia*: Capriva del Friuli, Dolegna del Collio, Mossa; *Udine*: San Giovanni al Natisone | Friuli-Venezia Giulia | Our interview |
| R4 | [Mais di Brumano] | *Bergamo*: Brumano, Zanica | Lombardia | Our interview |
| R5 | Nero spinoso | *Brescia*: Esine, Pertica Alta, Piancogno | Lombardia | Buffoli (2015); Regione Lombardia (2017); UNIMONT (2015); our interview |
| R6 | Nostrano di Pasiano | *Bergamo*: Songavazzo; *Pordenone*: Pasiano di Pordenone; *Venezia*: Noventa di Piave; *Vicenza*: Marano Vicentino | Friuli-Venezia Giulia; Lombardia; Veneto | Our interview |
| R7 | Pignoletto del Canavese | *Alessandria*: Spigno Monferrato; *Novara*: Gattico; *Torino*: Avigliana, Candia Canavese, Cantalupa, Cascinette d’Ivrea, Castellamonte, Cercenasco, Ciriè, Cumiana, Giaveno, Osasco, Pinerolo, Rivara, San Benigno Canavese, San Giorgio Canavese, Vische, Volvera; *Vercelli*: Palazzolo Vercellese | Piemonte | Associazione Antichi Mais Piemontesi (2017); Cascina Sala (2012); Consorzio Operatori Turistici Valli del Canavese (2018); CRAB (2004); Spagnolo et al. (2003) |
| R8 | Pignoletto della Val Cosa | *Pordenone*: Sequals | Friuli-Venezia Giulia | Our interview |
| R9 | Pignoletto [di Nervesa della Battaglia] | *Treviso*: Nervesa della Battaglia | Veneto | Our interview |
| R10 | Pignoletto [di Palmanova] | *Udine*: Palmanova and neighbouring municipalities | Friuli-Venezia Giulia | Azienda Agricola Lucia (2018); our interviews |
| R11 | Pignoletto giallo | *Cuneo*: Alba, La Morra; *Novara*: Galliate; *Torino*: Avigliana, Bosconero, Bussoleno, Campiglione Fenile, Candia Canavese, Castellamonte, Cercenasco, Giaveno, Moncalieri, Osasco, Romano Canavese, Vallo Torinese, Volvera | Piemonte | Associazione Antichi Mais Piemontesi (2017); Azienda Agricola Rabellotti Stefano (2018); CRAB (2004); Spagnolo et al. (2003) |
| R12 | Pignoletto rosso [del Medio Friuli] | *Udine*: Basiliano and neighbouring municipalities | Friuli-Venezia Giulia | Our interview |
| R13 | Rosso di Banchette | *Torino*: Banchette | Piemonte | Biocolture Banchette (2017); Farnè (2017) |
| R14 | Rosso di Brescia | *Cremona*: Pessina Cremonese; *Mantova*: Castel d’Ario | Lombardia | Azienda Agricola Porcalora (2018); Coldiretti (2014); Dilda (2018); our interviews |
| R15 | Rostrato di Cantello | *Varese*: Cantello | Lombardia | Anonym (2016, 2017); Bertolini (2002); Slow Food Provincia di Varese (2017) |
| R16 | [Rostrato di Mortara] | *Pavia*: Mortara | Lombardia | Our interview |
| R17 | Rostrato di Valchiavenna | *Sondrio*: Chiavenna, Gordona, Prata Camportaccio, Samolaco | Lombardia | Bertolini (2002); our interviews |
| R18 | Rostrato rosso di pianura | *Bergamo*: Albano Sant’Alessandro, Ambivere | Lombardia | Azienda Agrituristica Sant’Alessandro (2017); Ubaldi (2018); our interview |
| R19 | Rostrato rosso di Rovetta | *Bergamo*: Cene, Rovetta, Songavazzo | Lombardia | Provincia di Bergamo (2018); Regione Lombardia (2017); Rosso Mais (2017); Storie Enogastronomiche (2016) |
| R20 | Spin di Caldonazzo | *Trento*: Altopiano della Vigolana, Castello Tesino, Novaledo, Sant’Orsola Terme; *United States of America* | Trentino-Alto Adige | Azienda Agricola “Ai Masi” (2018); Azienda Agricola Belli Elena (2018); Azienda Agricola - Fattoria Didattica Maso da Tilio Bailo (2018); Bertolini, Franchi & Frisanco (2005); Ca’ dei Baghi (2017); Marchio Trentino (2013); Mas del Saro - Agriturismo, agricoltura naturale di montagna (2018); Nick’s Organic Farm (2018); Rubel (2009); Simonini (2014) |
| R21 | Spinato di Gandino | *Bergamo*: Casnigo, Cazzano Sant’Andrea, Gandino, Leffe, Peia | Lombardia | Commissione comunale per la tutela del territorio (De.C.O.) (2011); Comunità del Mais Spinato di Gandino (2018); Regione Lombardia (2017); Zanoletti (2015) |
| R22 | Spinusa nera | Donnas | Valle d’Aosta | Our interview |
| R23 | Sponcio | *Belluno*: Arsiè, Cesiomaggiore, Feltre, Fonzaso, Pedavena, San Gregorio nelle Alpi, Seren del Grappa | Veneto | Arduin & Sanson (2015); Bazolle (1987); Cooperativa agricola La Fiorita (2018); Veneto Agricoltura (2014) |
| R24 | Türc | *Sondrio*: Piateda | Lombardia | Our interview |
| R25 | Carlùn | *Sondrio*: Villa di Tirano | Lombardia | Our interview |
| R26 | Marano [del Lago d’Iseo] | *Sondrio*: Villa di Tirano | Lombardia | Our interview |
| R27 | [Rostrato di Sorico] | *Como*: Sorico | Lombardia | Our interview |
| R28 | [Rostrato giallo di Prata Camportaccio] | *Sondrio*: Prata Camportaccio | Lombardia | Our interview |

**Anonym. 2016.** L’antico mais rostrato si presenta al mulino. LaPrealpina.it. *Available at http://www.prealpina.it/pages/lantico-mais-rostrato-si-presenta-al-mulino-127796.html* (accessed 7 February 2018).

**Anonym. 2017.** Dopo l’asparago ecco il mais. LaPrealpina.it. *Available at http://www.prealpina.it/pages/nopermalink-137249.html* (accessed 11 January 2018).

**Arduin M, Sanson S. 2015.** Mais Sponcio: un esempio di biosviluppo per la montagna bellunese. Convegno Biodiversità nascosta. Valori e pratiche della diversità biologica nelle aree fragili. Consorzio Università Rovigo, 20−21 Mar 2015.

**Associazione Antichi Mais Piemontesi. 2017.** Antichi Mais Piemontesi. *Available at http://www.antichimaispiemontesi.it/* (accessed 21 December 2017).

**Azienda Agricola “Ai Masi”. 2018.** *Available at https://www.facebook.com/aziendaAiMasi/* (accessed 16 January 2018).

**Azienda Agricola Belli Elena. 2018.** Azienda Agricola Apicoltura Belli Elena. *Available at http://www.agribellielena.com/* (accessed 9 January 2018).

**Azienda Agricola - Fattoria Didattica Maso da Tilio Bailo. 2018.** *Available at https://www.facebook.com/aziendaagricolamasodatiliobailo/* (accessed 16 January 2018).

**Azienda Agricola Lucia. 2018.** *Available at http://www.aziendaagricolalucia.it* (17 January 2018).

**Azienda Agricola Porcalora. 2018.** *Available at https://www.facebook.com/porcalora/* (accessed 16 January 2018).

**Azienda Agricola Rabellotti Stefano. 2018.** *Available at https://www.rabellotti.it* (accessed 9 January 2018).

**Azienda Agrituristica Sant’Alessandro. 2017.** L’agriturismo Sant’Alessandro. *Avaliable at http://agriturismosantalessandro.it/* (accessed 20 February 2018).

**Bazolle M. 1987.** *Il possidente bellunese. Vol. II.* Feltre: Tipolitografia Beato Bernardino.

**Bertolini M. 2002.** *Mais in Lombardia: varietà tradizionali. Quaderni della ricerca*. Milano: Regione Lombardia; Bergamo: Istituto Sperimentale per la Cerealicoltura, Sezione di Bergamo.

**Bertolini M, Franchi R, Frisanco F. 2005.** *Il mais, una storia anche trentina*. San Michele all’Adige: Istituto Agrario di San Michele all’Adige.

**Ca’ dei Baghi. 2018.** Associazione per la Tutela e la valorizzazione della farina della Valsugana. *Available at http://www.cadeibaghi.com/cadeibaghi/presentazione/53.html* (accessed 9 January 2018).

**Cascina Sala. 2012.** Mais Pignoletto Rosso. *Available at http://www.cascinasala.it/mais.html* (accessed 11 January 2018).

**Coldiretti. 2014.** Nasce a Pessina Cremonese la polenta “favorita dal pubblico” al Festival di Levico Terme. Mondo Padano. *Available at http://www.mondopadano.it/stories/attualita/8522_nasce_a_pessina_cremonese_la_polenta_favorita_dal_pubblico_al_festival_di_levico_terme/#.WlNBv0tG38O* (accessed 8 January 2018).

**Commissione comunale per la tutela del territorio (De.C.O.). 2011.** *Disciplinare per la salvaguardia, caratterizzazione e valorizzazione della varietà locale di mais denominata “Spinato di Gandino”*. Gandino: Comune di Gandino (BG).

**Comunità del Mais Spinato di Gandino. 2018.** Mais Spinato di Gandino. *Available at* *http://www.mais-spinato.com* (accessed 10 January 2018).

**Consorzio Operatori Turistici Valli del Canavese. 2018.** Azienda Agricola Il Molino di Pero’o. *Available at http://www.turismoincanavese.it/it/prodotti-tipici-eccellenza/item/azienda-agricola-il-molino-di-pero-o.html* (accessed 16 Jan 2018).

**Cooperativa agricola La Fiorita. 2018.** *Available at http://www.cooperativalafiorita.it/?scheda_prodotto+prodotti=mais_sponcio* (accessed 10 January 2018).

**CRAB. 2004.** *Gli antichi mais del Piemonte*. Torino: Provincia di Torino.

**Dilda E. 2018.** Il Campagnino. *Available at https://www.agriturismoilcampagnino.it/* (accessed 8 January 2018).

**Farnè F. 2017.** Da Banchette il Mais rosso della tradizione. La Sentinella del Canavese. *Available at http://lasentinella.gelocal.it/tempo-libero/2017/12/22/news/da-banchette-il-mais-rosso-della-tradizione-1.16271716* (accessed 11 January 2018).

**Marchio Trentino. 2013.** Disciplinare di produzione della farina di mais da polenta. *Available at http://www.marchiotrentino.it/documenti/Marchio%20Qualit%C3%A0/disciplinari/disciplinare-farina-di-mais-2013.pdf* (accessed 6 February 2018).

**Mariani L. 2012.** Dal mais alla polenta. In: Mariani L, Pirovano M, eds. Il cibo e gli uomini. L’alimentazione nelle collezioni etnografiche lombarde. Galbiate: Rete Musei e Beni Etnografici Lombardi, Parco Monte Barro, Museo Etnografico dell’Alta Brianza, 59−69.

**Mas del Saro - Agriturismo, agricoltura naturale di montagna. 2018.** *Available at https://www.facebook.com/masdelsaro/* (accessed 16 January 2018).

**Nick’s Organic Farm. 2018.** Organic Red Floriani. “Spin Rossa della Valsugana”. *Available at http://www.nicksorganicfarm.com/images/NOF_Floriani_Info_Sheet.pdf* (accessed 3 February 2018).

**Provincia di Bergamo. 2018.** La biodiversità del mais tra tradizione e innovazione sulla tavola. *Available at http://www.provincia.bergamo.it/provpordocs/ROSSO.pdf* (accessed 6 February 2018).

**Regione Lombardia. 2017.** Schede descrittive delle varietà agrarie da conservazione. Ultimo aggiornamento 18/12/2017. *Available at http://www.regione.lombardia.it* (accessed 20 December 2017).

**Rosso Mais. 2017.** Rosso Mais. Mais Rostrato Rosso di Rovetta. *Available at http://www.rossomais.it/* (accessed 8 January 2018).

**Rubel W. 2009.** The Return of a Great Red Corn Variety. *Available at https://www.motherearthnews.com/real-food/red-corn-zmaz09fmzraw* (accessed 3 February 2018).

**Simonini, M. 2014.** Dal mais Spin di Caldonazzo nasce la farina della Valsugana. *Vita in Campagna* 11:62−63.

**Slow Food Provincia di Varese. 2017.** Cena – degustazione antico Mais Rostrato di Cantello. *Available at https://www.slowfoodvarese.it/2017/02/cena-degustazione-mais-rostrato-cantello* (accessed 8 January 2018).

**Spagnolo S, Pinna M, Gamba U, Zaccara P, Possetto D, Valoti P. 2003.** Valutazione di ecotipi piemontesi di mais da polenta in coltivazione biologica. *Bollettino di agricoltura biologica* 1:81−92.

**Storie Enogastronomiche. 2016.** Mais Rostrato di Rovetta, salvato da Giovanni Marinoni. *Available at https://www.youtube.com/watch?v=DO_qfNy9hzM* (accessed 8 January 2018).

**Ubaldi G. 2018.** La polenta con i mais autoctoni: dove assaggiarla a Bergamo. *Il Giornale del Cibo.* *Available at* *http://www.ilgiornaledelcibo.it/dove-mangiare-la-polenta-a-bergamo-grani-locali/* (accessed 21 March 2018).

**UNIMONT. 2015.** Intervista Saloni mais blu spinato 10 02 2015. *Available at* https://www.youtube.com/watch?v=UMmFK5cUcZU (accessed 20 December 2017).

**Veneto Agricoltura. 2014.** *Atlante dei prodotti agroalimentari tradizionali del Veneto*. Legnaro: Veneto Agricoltura.

**Zanoletti, C. 2015.** Mais Spinato di Gandino. Nascita e sviluppo di un progetto territoriale. Università degli Studi di Milano. Facoltà di Scienze Agrarie e Alimentari. Corso di Laurea in Valorizzazione e Tutela dell’Ambiente e del Territorio Montano. *Available at http://www.unimontagna.it/tesi/mais-spinato-di-gandino-nascita-e-sviluppo-di-un-progetto-territoriale/* (accessed 10 January 2018).
